# Effects of high-intensity interval exercise on arterial stiffness in individuals at risk for cardiovascular disease: a meta-analysis

**DOI:** 10.3389/fcvm.2024.1376861

**Published:** 2024-04-17

**Authors:** Ping Luo, Ruoshan Wu, Weifeng Gao, Weiyi Yan, Ruixue Wang, Yufang Ye

**Affiliations:** ^1^ School of Physical Education, Wuhan Sport University, Wuhan, China; ^2^ School of Physical Education, Hunan University of Science and Technology, Xiangtan, China

**Keywords:** high-intensity interval exercise, arterial stiffness, cardiovascular disease, pulse wave velocity, resting heart rate, augmentation index, systolic blood pressure, diastolic blood pressure

## Abstract

**Objective:**

The purpose of this meta-analysis was to investigate the effect of high-intensity interval training (HIIT) on arterial stiffness (AS) and vascular function in persons at high risk of cardiovascular disease (CVD).

**Methods:**

We conducted a comprehensive search of randomized controlled trials (RCTs) published in electronic databases (PubMed, Web of Science, Cochrane, Embase, and Ebsco) since their inception through October 2023 to evaluate the effect of HIIT on AS and vascular function in persons at high risk for CVD. The weighted mean difference (WMD) and 95% confidence intervals (95% CI) were calculated, and heterogeneity was assessed using the *I*^2^ test.

**Results:**

This study included 661 participants from 16 studies. HIIT significantly reduced pulse wave velocity (PWV) in persons at high risk for CVD [weighted mean difference (WMD), −0.62; 95% CI, −0.86–−0.38; *P* < 0.00001]. Subgroup analysis showed that the PWV improvement effect was better when the HIIT program was performed 2–3 times per week and the duration was controlled within 40 min [2–3 times, −0.67; 95% CI, −0.93–−0.41; *P* < 0.00001; time of duration, ≤40 min, −0.66; 95% CI, −0.91–−0.41; *P* < 0.00001]. HIIT significantly reduced systolic blood pressure (SBP, −5.43; 95% CI, −8.82–−2.04; *P* = 0.002), diastolic blood pressure (DPB, −2.96; 95% CI, −4.88–−1.04; *P* = 0.002), and resting heart rate (RHR, −4.35; 95% CI, −7.04–−1.66; *P* = 0.002), but had no significant effect on augmentation index (AIX, −2.14; 95% CI, −6.77–2.50; *P* = 0.37).

**Conclusion:**

HIIT can improve PWV in high-risk individuals with CVD and reduce SBP, DBP, and RHR, but has no significant effect on AIX. HIIT can effectively improve AS and vascular function and can be recommended as an effective method to improve AS in high-risk persons with CVD.

**Systematic Review Registration:**

https://www.crd.york.ac.uk/PROSPERO/, identifier: CRD42023471593.

## 1 Introduction

Cardiovascular disease CVD is one of the leading causes of mortality worldwide and a major risk factor for morbidity worldwide (1). Therefore, it is necessary to reduce the incidence and risk factors of CVD, as physical activity is known to reduce mortality from CVD (2). Changes in CVD risk factors, such as body weight, blood pressure, and lipids, explain 60% of the beneficial effects of exercise on major CVD outcomes, and the remaining 40% of the reduction in risk factors is associated with vascular hemodynamics (3, 4). Arterial stiffness (AS) is a strong independent predictor of all-cause mortality owing to cardiovascular events (5–8). Increased AS can lead to increased blood pressure, left ventricular hypertrophy, decreased ventricular diastolic function, coronary ischemic disease, and decreased sensitivity to arterial baroreflex (9–12). Therefore, preventing CVD by reducing the incidence of AS is essential.

Pulse wave velocity (PWV) is the gold standard for measuring AS, especially carotid-femoral pulse velocity (CF-PWV) (13). An increase of 1 m/s in PWV is associated with 14% and 15% increased risk of cardiovascular events and mortality, respectively (5). Therefore, finding effective interventions to reduce PWV is one of the main goals for preventing CVD and improving cardiovascular function (14, 15).

Exercise improves blood vessel function, and many meta-analyses on exercise and cardiovascular function have shown that appropriate exercise can effectively improve AS in different populations (16–20). Studies on the effect of exercise on AS mainly focus on sustained aerobic exercise. Previous meta-analyses have shown that continuous aerobic exercise is effective in reducing AS in hypertensive, elderly, and obese populations (16, 17, 21, 22). Although sustained aerobic exercise has many benefits in terms of improving blood vessel function, there are also some drawbacks; for example, participants tend to have an aversion to exercise due to the monotony of exercise forms, which reduces the beneficial effects of exercise (23). Conversely, HIIT has been shown to be equally or even better at stimulating health benefits than moderate-intensity continuous training (MICT) and is considered a time-saving aerobic exercise (24, 25). Additionally, HIIT has a higher level of compliance than MICT, and the benefits of exercise are even higher (25–28). Therefore, HIIT can be used as an effective alternative to continuous aerobic exercise (25–28). Previous studies have shown that resistance training has no harmful effects on AS in individuals at high risk of CVD (29). However, the effect of HIIT on AS in persons at a high risk of CVD or in those carrying high-risk factors for CVD is inconclusive.

Therefore, this study aimed to conduct a comprehensive systematic review and meta-review of randomized controlled trials (RCTs) to explore whether HIIT improves PWV, augmentation index (AIX), SBP, DBP, and RHR in persons at high risk for CVD. In addition, subgroup analyses were performed to examine whether HIIT intervention programs affected the effect of PWV improvement. The results of this study will provide theoretical references and suggestions for the exercise program of high-risk groups with CVD in the future.

## 2 Materials and methods

### 2.1 Trial registration

This systematic review and meta-analysis followed the PRISMA (Preferred Reporting Program for Systematic Reviews and Meta-Analysis) guidelines (30). The study topics and proposals were registered with PROSPERO (CRD42023471593).

### 2.2 Search strategy

Two experienced researchers (PL and RSW) applied the PICOS principles (subjects, interventions, comparisons, outcomes, and study design) to search electronic databases (PubMed, Web of Science, Cochrane, Embase, and Ebsco) from their inception until October 2023. For searches in PubMed/Cochrane, and Embase, terms from MeSH and Emtree were used, respectively. Search formula: (“High intensity interval training” OR “Interval training” OR “Aerobic interval training” OR “Combination training” OR “Intermittent training”) AND (“Arterial stiffness” OR “Vascular stiffness” OR “Aortic stiffness” OR “Pulse wave velocity” OR “Augmentation index”). Supplementary Table S1 provides specifics detail of the search method that was used for each database. If there was any disagreement between the two authors, a third author (WFG) participated in the discussion until a consensus was reached.

### 2.3 Eligibility criteria

Articles included in the analysis must meet the following criteria: (1) Randomized controlled trials of HIIT vs. no exercise, usual care, or sedentary; (2) HIIT program lasting at least 6 weeks or a combination of HIIT and other forms of training; (3) According to the American College of Sports Medicine (ACSM) guidelines,participants have CVD, have CVD risk factors, or are individuals with high levels of circulating pro-inflammatory factors; (4) Provide at least one central PWV or peripheral PWV outcome measure; and (5) The article is written in English. Interval exercise can be broadly defined as a short to moderate period (10 s to 5 min) of repetitive training sessions with an intensity above the anaerobic threshold, with intervals of low-intensity activity or rest between training sessions, usually without complete recovery (31, 32). According to the ACSM guidelines, high intensity is defined as a person's estimated >80%HR max (33), >65%VO2 peak, >60%HR reserve/VO2 reserve, or >14 Borg rating of perceived exertion (RPE), this work also includes sprint interval training, which is usually performed at “all out” extreme intensity for relatively short periods of time (34). According to the above criteria, the literature meeting the criteria is included in this paper.

### 2.4 Data extraction

Two researchers (PL and RSW) independently used the same standardized tables created in Microsoft Excel for data extraction, and any disagreements were resolved through discussion with another researcher (WFG). Information extracted from eligible articles included: (1) Document characteristics (author, publication year, country); (2) Participant characteristics (sample size, age, proportion of women, disease status/risk factors, and medication use); (3) Intervention characteristics (exercise type, exercise cycle, duration, exercise intensity, exercise frequency, total exercise change, and interval time); and (4) Outcome measures. If the data is missing, the researcher (PL) contacts the original author by e-mail to obtain the missing data.

### 2.5 Quality assessment and sensitivity analyses

The Physiotherapy Evidence Database tool (PEDro) was used to evaluate the study quality. Studies with scores ≥6 were considered high quality, 4–5 were average quality, and <4 were low quality. The rating scale contained 11 items as follows: (1) The inclusion conditions of the subjects were clear; (2) Random allocation; (3) Grouping blind; (4) The main prognostic indicators were consistent at baseline; (5) The subjects were blind; (6) Training blind; (7) Blind evaluators for at least one major outcome; (8) More than 85% of the subjects were measured for at least one major outcome; (9) Subjects received treatment or control conditions according to the assignment plan; (10) Report intergroup statistical results for at least one major outcome; (11) Provide point measurements and variation measurements for at least one major result. Two reviewers (PL and RSW) independently assessed methodological quality. We excluded blind entries from the original 11-item scale (group blinds, subject blinds, training blinds, and all evaluators of at least one major outcome blinds). This is because participants' control over blinding conditions cannot be guaranteed during the intervention. Evaluation disagreements among reviewers are resolved by discussing and reaching a consensus with the third author (WFG). Supplementary Table S1 provides specific quality assessment details. A sensitivity analysis was performed by excluding each study individually to determine whether the results changed.

### 2.6 Statistical analysis

Anterior and posterior mean and SD values for PWV, AIX, SBP, DBP, and RHR were extracted from each study, and we calculated the change value of the data before and after the intervention to achieve the combined effect to make the forest map. The Q statistic was used to determine the inter-study heterogeneity, and statistical significance was set at *P* < 0.1. *I*^2^ values were used to evaluate heterogeneity. *I*^2^ values of 0%, 25%, 50%, and 75% indicated no heterogeneity, mild heterogeneity, moderate heterogeneity, and high heterogeneity, respectively. When heterogeneity is low, the fixed effects model is used to combine the data, in any case, the random effects model is used. In the subgroup analysis, we conducted a subgroup analysis of subjects' characteristics (male-female ratio, body mass index, and medication situation), interval intervention characteristics (total exercise change, exercise cycle, exercise duration, and interval duration), and baseline characteristics to explore the effect of different conditions on PWV. Analysis results and forest maps were generated using Review Manager (RevMan) 5.3 (The Cochrane Collaboration, Copenhagen, Denmark, 2019) software. Overall, *P* < 0.05 was considered statistically significant. Stata17.0 (Stata Corporation, College Station, TX; US) software was used to generate funnel plots, visual tests of Begg's tests were performed to discuss publication bias, and statistical significance was set at *P* < 0.1.

## 3 Results

### 3.1 Study selection

**Figure 1:**
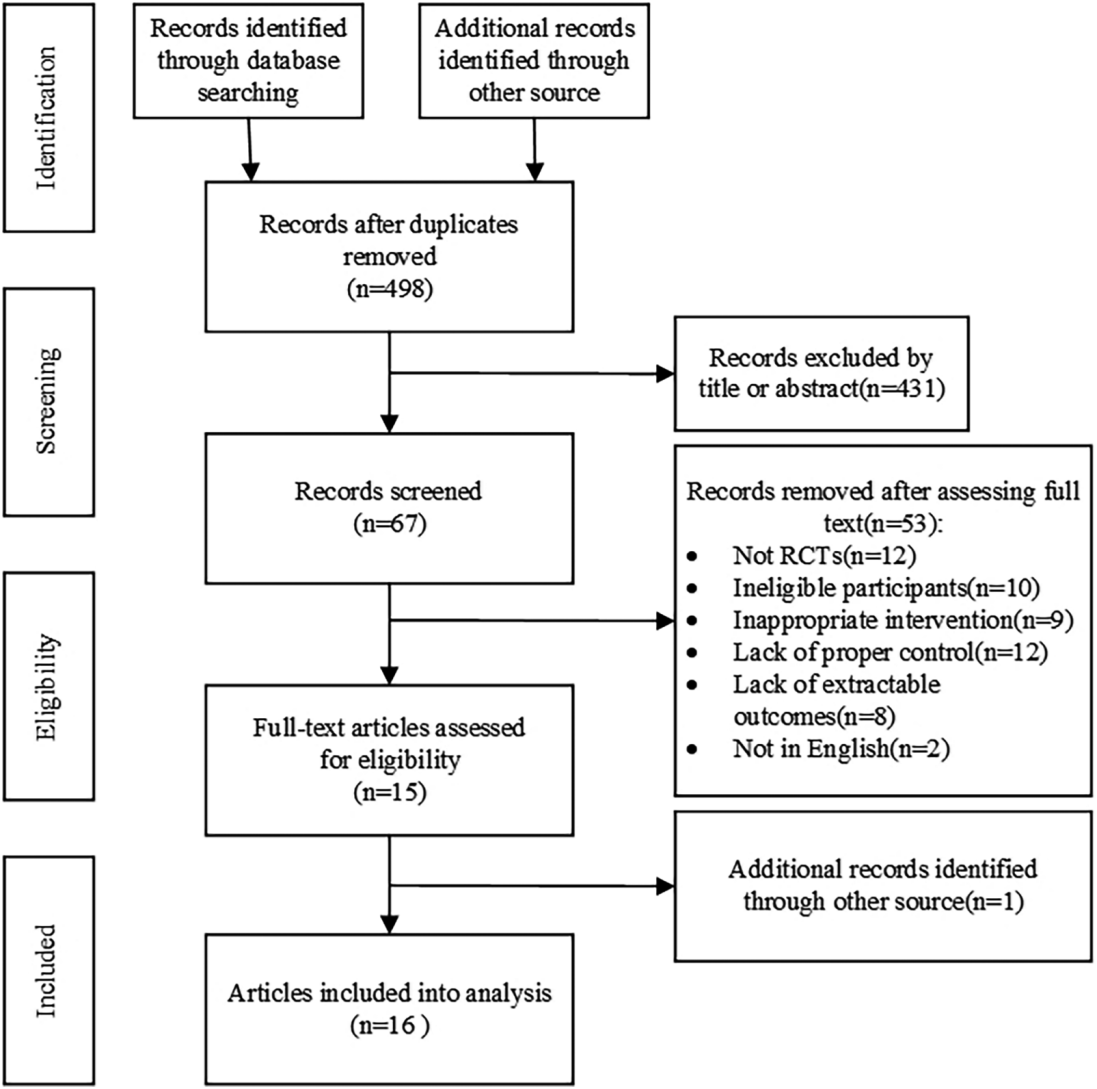
Flow diagram of systematic literature article search.

Figure 1 summarizes the research process for screening eligible conditions; 1,009 records were retrieved. After deleting duplicate literature, 67 records were found to be suitable by screening the titles and abstracts of the remaining literature; full-text screening was performed, and a total of 15 eligible references were obtained, and 1 was retrospectively obtained by other means. Sixteen studies were included in the meta-analysis. Fifty-three articles were excluded for the following reasons: not a randomized controlled trial (*n* = 12); subjects did not match (*n* = 10); the experimental intervention did not match (*n* = 9); the control group did not conform (*n* = 12); outcome index did not meet (*n* = 8); and language did not match (*n* = 2).

### 3.2 Characteristics of included studies

After screening, 16 studies and 18 research reports were included in this meta-analysis, among which two studies were extracted from the literature by Chuensiri et al. (35) and Bahmanbeglou et al. (36). The 18 research reports had a total sample size of 656, with 347 participants in the experimental group and 314 participants in the control group (Table 1).

**Table 1:** Characteristics of studies included in this meta-analysis.

Ref.	Country	Population	Medication	Exp/Con	Age (±SD)	Females (%)	Training total	Type	Interval time(s)	Intervention	Duration (Min)	Length (Weeks)	Frequency	Outcome	Quality
Guimarães et al. (37)	Brazil	Hypertension	Y	16	45 ± 9	25	Same	R	120	50%–80% HRR	40	16	3	①④	6
				11	47 ± 6	18									
Ciolac et al. (38)	Brazil	High familial risk for hypertension	N	16	24 ± 4	100	Same	W/R	120	50%–90% VO_2_ peak	40	16	3	①④	6
				12	25 ± 4										
Chrysohoou et al. (39)	Greece	Chronic heart failure	Y	33	63 ± 9	12	Increase	C	30	80%–100% WR peak	45	12	3	①②⑤	6
				39	56 ± 11	28				RT:30%–90%1RM					
Van Craenenbroeck et al. (40)	Belgium	Chronic kidney disease	Y	19	52 ± 12	42	Same	C	N/R	90% HR max	40	12	7	①②⑤	6
				21	55 ± 14	48									
Chuensiri et al. (35)	Thailand	Obese	N	15	11 ± 0	0	Same	C	10	170% Peak power output	15	12	3	①④⑤	6
				11	11 ± 0				60		35				
				11	11 ± 0					90% Peak power output					
KIM et al. (41)	American	Older adults	Y	14	65 ± 1	76	Same	C	180	70%–90% HR max	40	8	4	①④⑤	6
				11	63 ± 2	71									
Bellia et al. (42)	Italy	Type 2 diabetic	Y	11	59 ± 8	18	Increase	W	180	45%–80% HR max	45	12	2–3	①③	6
				11	56 ± 6	36									
Mora-Rodriguez et al. (43)	Spain	Metabolic syndrome	N	23	53.5 ± 8.9	17	Same	C	180	70%–90% HR max	40	24	3	①③④	6
				23											
Hanssen et al. (44)	Switzerland	Migraine	N	13	36 ± 11	77	Same	R	180	90%–95% HR max	45	12	2	①②③④	6
				12	37 ± 12	83									
Bahmanbeglou et al. (36)	Iran	Stage 1 hypertension	Y	10	48 ± 5	N/R	Increase	W/R	30	80%–100% VO_2_ peak	short: 42	8	3	①④	6
				10	49 ± 5				240	75%–90% VO_2_ peak	long: 47				
				10	47 ± 3										
Bouaziz et al. (45)	France	Older adults	Y	30	73 ± 3	70	Same	C	60	40%–100% VT1	30	9.5	2	①④	6
				30	74 ± 3	77									
Deiseroth et al. (46)	Switzerland	Older adults	Y	38	58 ± 5	47	Same	W	180	60%–90% HR max	60	12	3	①④⑤	5
				30	57 ± 6	63									
Ho et al. (47)	Australia	Post-menopausal women	N	30	54 ± 4	100	Same	C	12	50–120 RPM	30	8	3	①④⑤	5
				30	53 ± 3										
Way et al. (48)	Australia	Type 2 diabetes	Y	12	57 ± 2	42	Same	C	N/R	50%–90% VO_2_peak	19	12	3	①②③④	6
				11	52 ± 1	36									
McNarry et al. (49)	UK	Asthma	Y	16	14 ± 1	N/R	Same	G	10–30	90% HR max	30	24	3	①②④⑤	6
				17											
TAHA et al. (50)	Saudi Arabia	Obese hypertensive	Y	30	48 ± 4	100	Increase	C	180	60%–90% HR max	40	12	3	①③④	6
				30	49 ± 4										

N/R, not reported; R, running; C, cycling; W, walking; G, gaming; HRR, heat rate reserve; WR peak, limit of tolerance; RT, resistance training; HR max, heart rate maximum; VT1, first ventilatory threshold; RM, repetition maximum; RPM, revolutions per minute. ①, pulse wave velocity; ②, augmentation index; ③, augmentation index corrected for 75 beats per min; ④, peripheral blood pressure; ⑤, resting heart rate.

Participants ranged in age from 11–75 years, with middle-aged and elderly studies accounting for the majority. 11 studies included medication use during the intervention period (36, 37, 39–42, 45, 46, 48–50), and 5 studies explicitly indicated no medication treatment during the intervention period (35, 38, 43, 44, 47). 6 studies included women with a female ratio of >70 percent (38, 41, 44, 45, 47, 50), three of which had all-female participants (38, 47, 50), and 2 studies did not report a sex ratio (36, 49). High-intensity interval training was used in all the included studies, which included 4 incremental exercises (36, 39, 42, 50), and the rest were equal exercises (35, 37, 38, 40, 41, 43–45, 47–49). 9 studies used bicycle intervention (35, 39–41, 43, 45, 47, 48, 50), 6 studies used treadmill intervention (36–38, 42, 44, 46), and 1 study used a game form (49). The intermittent time ranged from 10–240s, and most of the single intervention times were approximately 40 min. Except for KIM et al. (41) and Van Craenenbroeck et al. (40), 2 studies intervened 4 and 7 times per week, respectively, and the other studies intervened 2–3 times per week, with an intervention cycle greater than or equal to 8 weeks. PWV was tested in 16 studies,of which 10 used carotid-femoral PWV to assess AS (37–41, 43, 45, 46, 48, 49), and 1 used central aortic PWV to in review assess AS (44). 3 studies provided brachial-ankle PWV (35, 47, 50), 2 studies provided carotid-radial PWV (42, 45), and 1 study did not report specific AS assessment methods (36). In the subgroup analysis, we classified the carotid-femoral PWV and central aortic PWV as the central PWV for statistical analysis, while the brachial-ankle PWV and the carotid-radial PWV were divided into peripheral PWV for data analysis. 5 articles provided AIX (39, 40, 44, 48, 49) and 5 provided AIX-75 (42–44, 48, 50). Brachial arterial blood pressure was reported in 13 studies (35–38, 41, 42, 44–50). RHR before and after the intervention was reported in 7 studies (35, 39–41, 46, 47, 49).

### 3.3 Risk of bias

**Figure 2:**
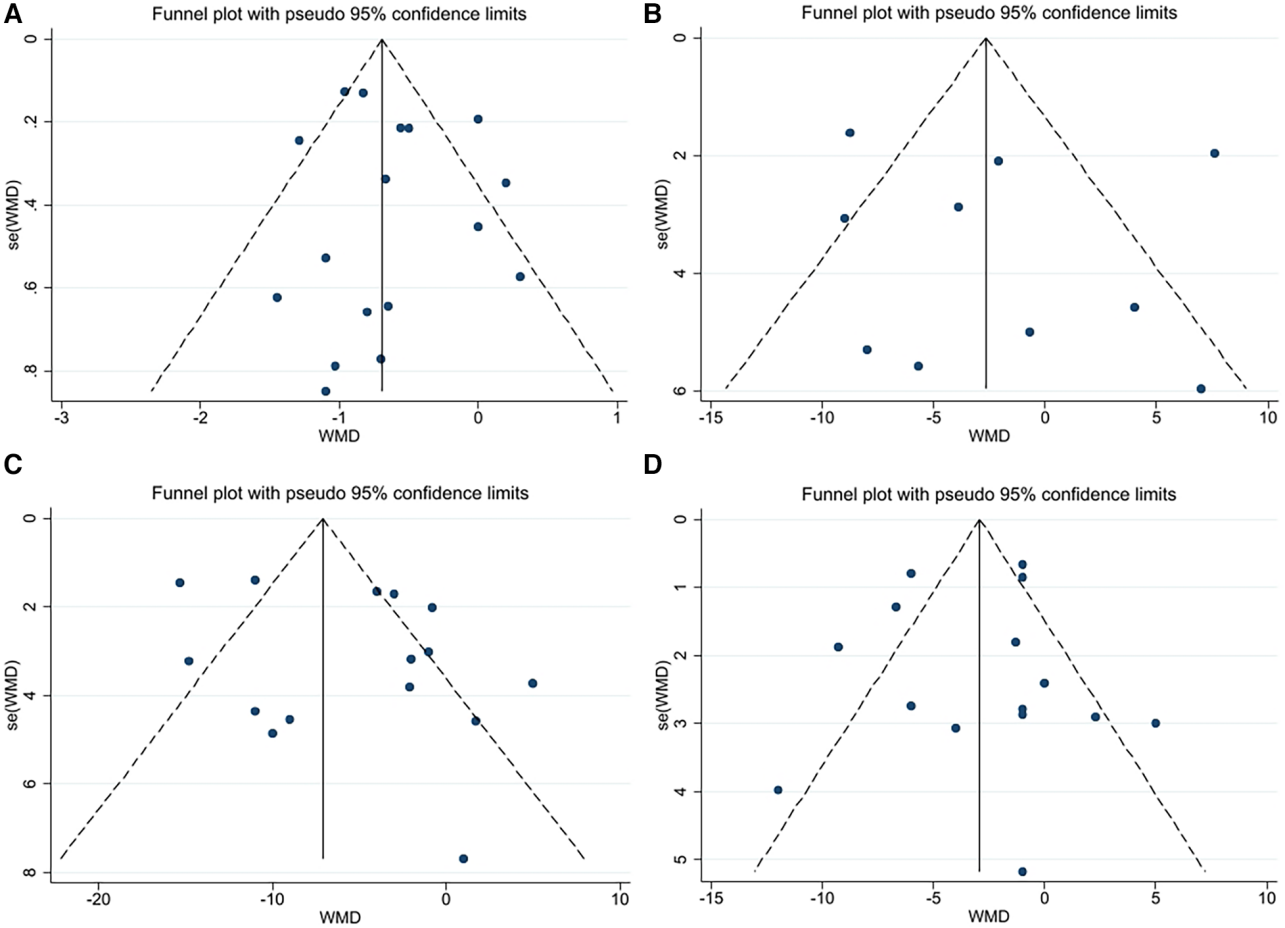
(**A**) Funnel plot for PWV. (**B**) Funnel plot for AIX. (**C**) Funnel plot for SBP. (**D**) Funnel plot for DBP.

A funnel plot was constructed using Stata software to visually assess the risk offset (Figure 2). Since there were less than ten samples of RHR, a funnel plot was not drawn.

### 3.4 Results of meta-analysis

#### 3.4.1 Effect of high-intensity interval exercise on pulse wave velocity

**Figure 3:**
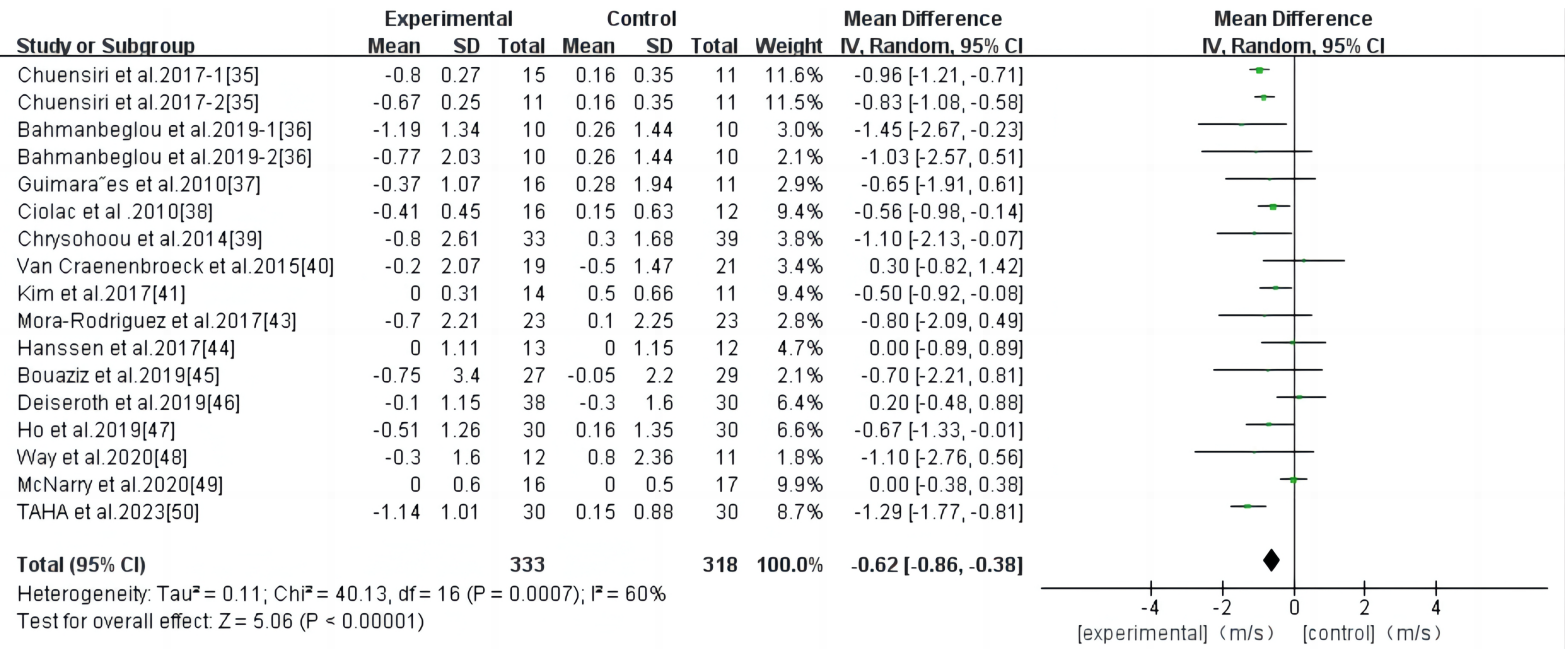
Meta-analysis results of high-intensity interval exercise on PWV.

Eighteen studies were included in the analysis, and the results of the meta-analysis (Figure 3) showed a high degree of heterogeneity among the studies (*I*^2 ^= 83%, *P* < 0.00001). Using the random effects model, the total effect size of HIIT significantly reduced PWV (WMD, −0.77; 95% CI, −1.11–−0.43]. Sensitivity analysis was used to exclude the included studies individually and assess the impact of each study on the PWV effect size. After the exclusion of the study by Bellia et al. (42), the heterogeneity was significantly reduced (*I*^2 ^= 60), while the combined effect size was slightly reduced (WMD, −0.62; 95% CI, −0.86–−0.38), the results remained statistically significant (*P* < 0.00001); therefore, the combined analysis of this study was excluded. Begg and Egger's tests showed that there was no significant bias in the included studies (*P* > 0.1).

#### 3.4.2 Effect of high-intensity interval exercise on augmentation index

**Figure 4:**
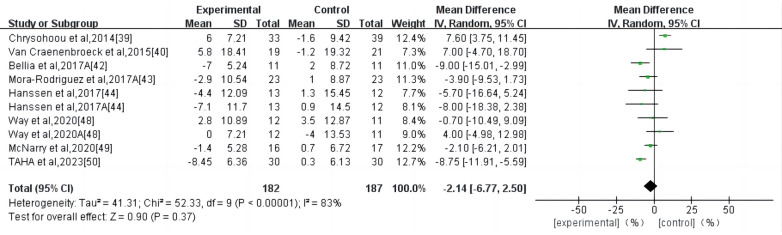
Meta-analysis results of high-intensity interval exercise on AIX. (**A**) AIX for 75 beats per minute.

HIIT had no significant effect on the AIX in the high-risk individuals for CVD (WMD, −2.14; 95% CI, −6.77–2.50; *P *= 0.37) (Figure 4). There was a high heterogeneity among the studies (*I*^2 ^= 83%). Begg and Egger's tests showed no significant bias in the included studies (*P* > 0.1).

#### 3.4.3 Effect of high-intensity interval exercise on systolic blood pressure

**Figure 5:**
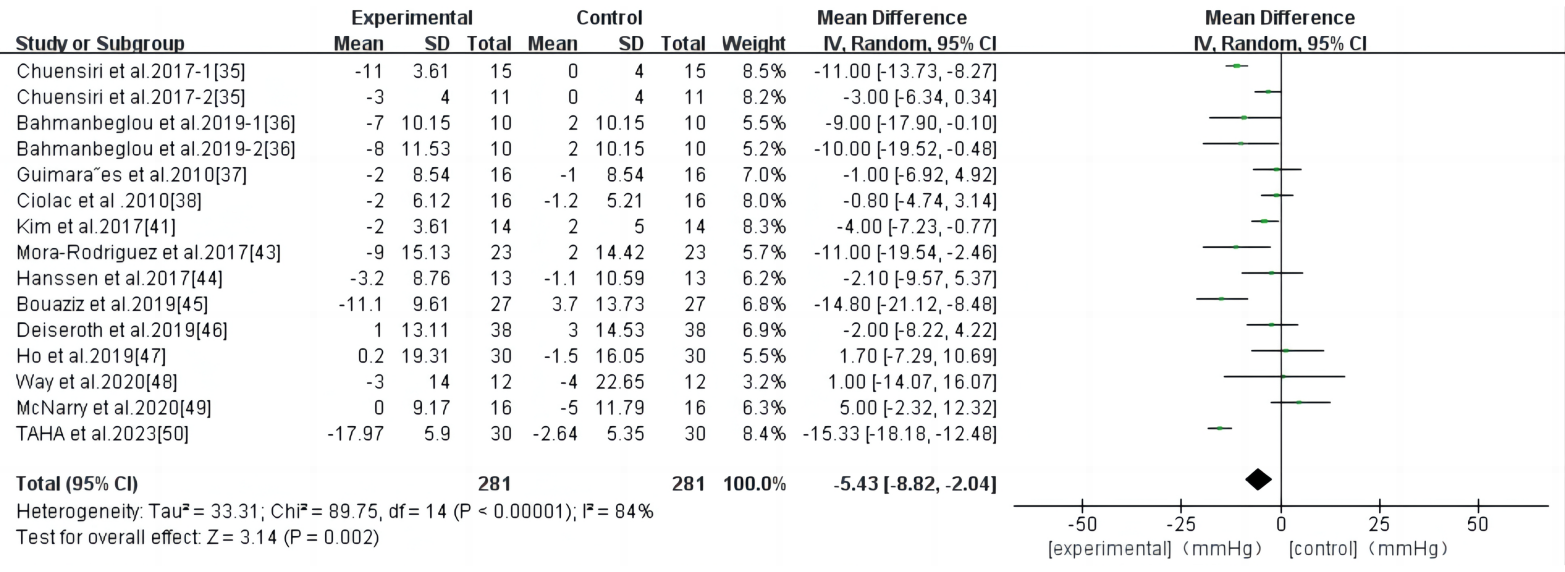
Meta-analysis results of high-intensity interval exercise on SBP.

HIIT had a significant effect on SBP in persons with high-risk CVD (WMD, −5.43; 95% CI, −8.82–−2.04; *P* = 0.002) (Figure 5). There was a high heterogeneity among the studies (*I*^2 ^= 84%). Begg and Egger's tests showed no significant bias in the included studies (*P* > 0.1).

#### 3.4.4 Effect of high-intensity interval exercise on diastolic blood pressure

**Figure 6:**
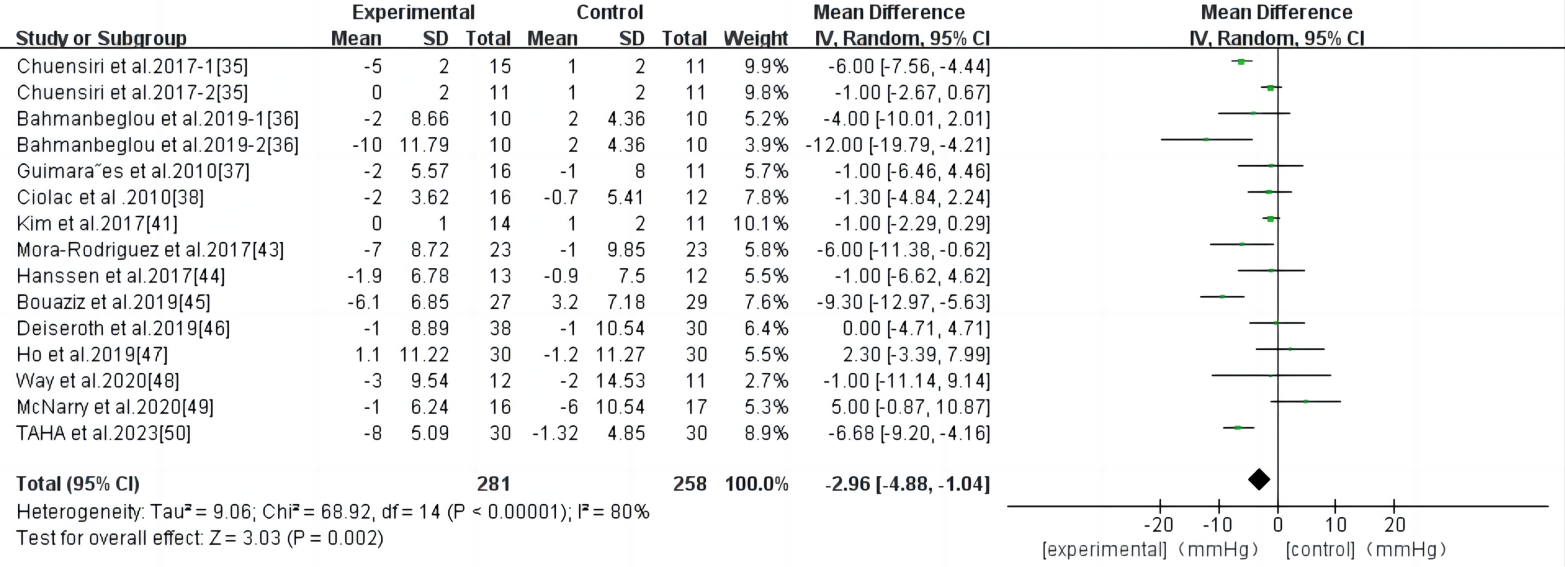
Meta-analysis results of high-intensity interval exercise on DBP.

HIIT had a significant effect on DBP in persons with high-risk CVD (WMD, −2.96; 95% CI, −4.88–−1.04; *P* = 0.002) (Figure 6). There was significant heterogeneity among the studies (*I*^2 ^= 80%). Begg and Egger's tests showed no significant bias in the included studies (*P* > 0.1).

#### 3.4.5 Effect of high-intensity interval training on resting heart rate

**Figure 7:**
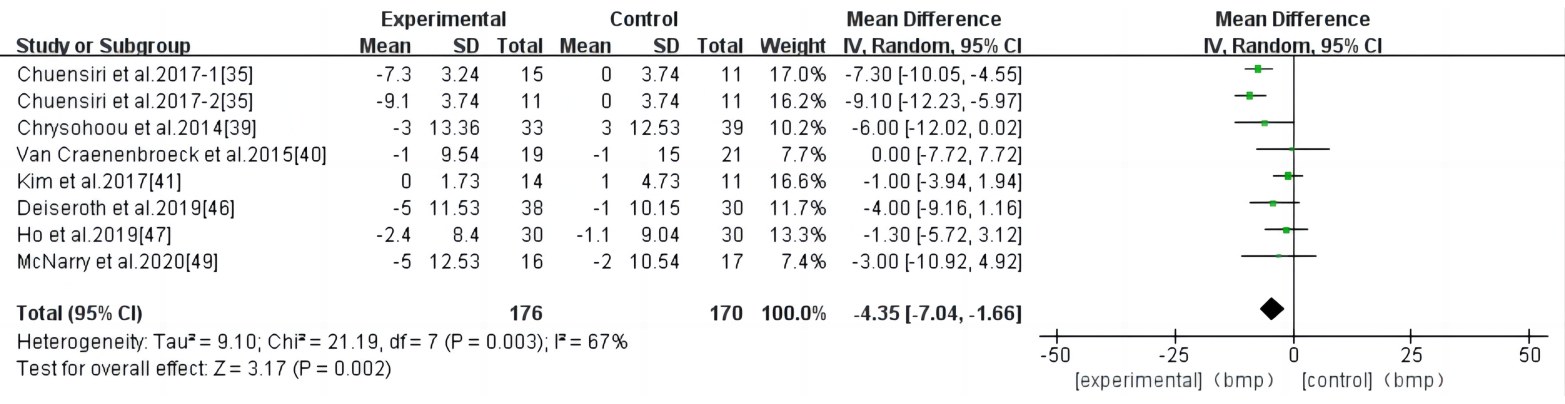
Meta-analysis results of high-intensity interval exercise on RHR.

HIIT had a significant effect on RHR in individuals at a high risk of CVD (WMD, −4.35; 95% CI, −7.04–−1.66; *P* = 0.002) (Figure 7). There was a high heterogeneity among the studies (*I*^2 ^= 67%). Begg and Egger's tests showed no significant bias in the included studies (*P* > 0.1).

#### 3.4.6 Subgroup analysis

A subgroup analysis was performed based on the PWV measurement location, baseline blood pressure, participant characteristics (age, medication use, and body mass index), and exercise intervention characteristics (total exercise change, exercise cycle, exercise frequency, exercise duration, and interval time). Data from the study by Bellia et al. (42) were excluded and are summarized in Table 2.

**Table 2:** Subgroup analyses assessing potential moderating factors for PWV in studies included in the meta-analysis.

Group	Studies		PWV(m/s)				
	Number	References	WMD (95% CI)	*I* ^2^	*P* overall change	*P* for sub dif	*P* for m
PWV assessment site							
Central PWV	11	(37–41, 43–46, 48, 49)	−0.32[−0.57–−0.08]	21	0.01	<0.0001	0.002
Peripheral PWV	4	(35, 45, 47, 50)	−0.93[−1.09–−0.77]	0	<0.00001		
Baseline SBP							
≥140	1	(50)	−1.29[−1.77–−0.81]	0	<0.00001	0.04	
130–139.9	8	(35, 37, 38, 41, 44, 47–49)	−0.58[−0.84–−0.31]	64	<0.00001		0.222
<130	4	(36, 43, 45, 46)	−0.62[−1.32–−0.09]	43	0.09		
Change SBP							
≥5	5	(35, 36, 43, 45, 50)	−1.03[−1.24–−0.82]	0	<0.00001	0.0008	0.015
<5	9	(35, 37, 38, 41, 44, 46–49)	−0.43[−0.71–−0.14]	59	0.004		
Baseline DBP							
≥90	1	(50)	−1.29[−1.77–−0.81]	0	<0.00001	0.03	
80–89.9	5	(36, 43, 45, 46, 48)	−0.65[−1.27–−0.03]	34	0.04		0.195
<80	7	(35, 37, 38, 41, 44, 47, 49)	−0.57[−0.84–−0.29]	68	<0.0001		
Change DBP							
≥5	5	(35, 36, 43, 45, 50)	−1.02[−1.23–−0.80]	0	<0.00001	0.003	0.033
<5	10	(35–38, 41, 44, 46–49)	−0.47[−0.76–−0.18]	58	0.001		
Training total							
Increase	3	(36, 39, 50)	−1.26[−1.66–−0.87]	0	<0.00001	0.001	0.026
Same	12	(35, 37, 38, 40, 41, 43–49)	−0.49[−0.75–−0.23]	61	0.0002		
Period							
≥12	11	(35, 37–40, 43, 44, 46, 48–50)	−0.58[−0.88–−0.27]	71	0.0002	0.78	0.587
<12	4	(36, 41, 45, 47)	−0.64[−0.97–−0.32]	0	0.0001		
Frequency							
2–3	13	(35–39, 43–50)	−0.67[−0.93–−0.41]	61	<0.00001	0.3	0.325
>3	2	(40, 41)	−0.28[−0.98–−0.43]	41	0.44		
Time of duration							
≤40	11	(35, 37, 38, 40, 41, 43, 45, 47–50)	−0.66[−0.91–−0.41]	62	<0.00001	0.75	0.582
>40	4	(36, 39, 44, 46)	−0.54[−1.23–−0.14]	56	0.12		
Interval time							
≥60	10	(35–38, 41, 43–46, 50)	−0.63[−0.91–−0.35]	48	<0.0001	0.74	0.795
<60	5	(35, 36, 39, 47, 49)	−0.73[−1.27–−0.19]	79	0.008		
Age							
<30	3	(35, 38, 49)	−0.61[−1.00–−0.22]	84	0.002	1	
30–59.9	9	(36, 37, 40, 43, 44, 46–48, 50)	−0.61[−1.05–−0.16]	55	0.008		0.863
≥60	3	(39, 41, 45)	−0.59[−0.97–−0.21]	0	0.002		
Body mass index							
≥30	5	(39, 43, 46, 48, 50)	−0.78[−1.50–−0.06]	69	0.03	0.04	
25–29.9	7	(35–37, 40, 41, 45, 47)	−0.80[−0.97–−0.63]	7	<0.00001		0.121
<25	3	(38, 44, 49)	−0.22[−0.64–−0.19]	51	0.29		
Sex (Female%)							
≥70	6	(38, 41, 44, 45, 47, 50)	−0.67[−1.01–−0.33]	47	0.0001	1	0.962
<70	7	(35, 37, 39, 40, 43, 46, 48)	−0.67[−0.99–−0.34]	51	<0.0001		
Medication							
Yes	10	(36, 37, 39–41, 45, 46, 48–50)	−0.58[−0.99–−0.17]	64	0.005	0.38	0.616
No	5	(35, 38, 43, 44, 47)	−0.78[−0.97–−0.59]	19	<0.00001		

PWV, pulse wave velocity; WMD, weighted mean difference; *P* for sub dif, *p* for subgroup difference; *P* for *m*, *p* value for the meta-regression analyses between subgroups.

## 4 Discussion

To our knowledge, this is the first meta-analysis of the effects of HIIT on AS and vascular function in persons with or carrying risk factors for CVD. This meta-analysis suggests that HIIT is effective in improving PWV in persons with CVD or risk factors for CVD. Performing HIIT 2–3 times/week for ≤40 min optimizes PWV improvement. HIIT also significantly improved SBP, DBP, and RHR in persons with CVD or risk factors for CVD but did not affect the AIX.

PWV is effective in assessing AS based on ultrasound measurements (51). The mechanisms leading to increased AS are complex, and oxidative stress and inflammation are the main causes of arteriosclerosis (52, 53). PWV is also associated with endothelial cells and smooth muscle function (54, 55). In addition, various substances and hormone levels in the body can affect AS, including nitric oxide (56–58), vasoconstrictors (59), advanced glycosylation end products (60), oxidized low-density lipoprotein (61), and aldosterone (62). Clinical studies have demonstrated that exercise can promote the production of nitric oxide and enhance its bioavailability to reduce AS (58). Published meta-analyses have shown a positive dose-response relationship between exercise intensity and AS improvement (18). This means that higher-intensity exercise may have a better effect on AS. Meanwhile, the physical activity guidelines issued by the ACSM recommend high-intensity exercise to maintain and improve cardiovascular health (63). Published meta-analyses have shown that aerobic and combined exercises (aerobic exercise combined with resistance) can effectively improve PWV in different populations (16–22). Consistent with previous findings, we showed that HIIT improved AS in persons with CVD or those at high risk for CVD (−0.62 m/s). According to the meta-analysis of Li et al. (17), long-term aerobic training can significantly reduce PWV (−0.75 m/s) in middle-aged and elderly people. A meta-analysis of adults aged ≥18 years by Ashor et al. (19) found that aerobic exercise significantly reduced PWV (−0.63 m/s). Zhang et al. (20) concluded that aerobic training significantly reduced cf-PWV in CVD population, and the result was similar to ours (−0.42 m/s vs. −0.32 m/s). The above evidence suggests that HIIT has a similar and more time-saving effect in improving PWV compared to MICT. While a small percentage of the interval literature we included would have been included in a meta-analysis of related aerobic exercise, we are the first to conduct a meta-analysis of HIIT with individuals who carry or have CVD risk factors, as well as a subgroup analysis of factors, such as intermittent exercise program and subject characteristics.

In the subgroup analysis of baseline blood pressure and pre and post intervention blood pressure changes, we observed that, while most groups achieved significant levels, the higher the baseline blood pressure or the greater the pre- and post-training blood pressure change, the more significant the PWV reduction. A published meta-analysis of aerobic exercise in hypertensive populations corroborates our results (17, 20). Montero et al. (64) concluded that aerobic training does not reduce AS in patients with (prehypertension) unless SBP is significantly reduced, or its duration is prolonged. This conclusion was confirmed by subgroup analysis of blood pressure changes (≥5) before and after HIIT intervention. Previous studies have also shown that PWV is highly correlated with blood pressure (65). A subgroup analysis of HIIT programs showed that HIIT performed 2–3 times per week for no more than 40 min resulted in a significant improvement in PWV. First, 90% of the studies were conducted two to three times a week and lasted ≤40 min. Therefore, it also weakens the strength of the evidence for this result. Second, although HIIT is better than MICT in terms of improving the heart, cardiovascular, and metabolic conditions, and HIIT can produce exercise enjoyment and exercise compliance similar to or better than MICT (66), it does not mean that longer or more frequent HIIT can achieve better exercise results, especially in CVD people. ACSM emphasizes that reasonable exercise intensity and frequency are more conducive to the recovery of the body, and also recommends HIIT once a week, and then increase the intensity and frequency of exercise when the body gradually to the intensity (33). This also reasonably explains why incremental training improved PWV better (−1.26 m/s vs. −0.49 m/s) than the same amount of training in the total training variation subgroup. We also found that PWV improved better in overweight or obese people who had or carried risk factors for cardiovascular disease. Studies have shown that excessive adipose tissue can produce a variety of the pro-inflammatory cytokines interleukin-6 (IL-6), tumor necrosis factor-α (TNF-α) and plasminogen activator inhibitor, which leads to inflammation and deterioration of arterial stiffness (67). At the same time, inflammation and oxidative stress caused by obesity inhibit the maturation and secretion of adiponectin (68), and excess fat in the body reduces adiponectin levels, further exacerbating metabolic disorders (69, 70). Experimental studies have shown that adiponectin plays a decisive role in energy metabolism, anti-inflammatory, and cardiovascular health (71). Effective inhibition of adipose tissue during HIIT may be a reasonable explanation for this result.Numerous studies have shown that HIIT is effective in reducing body weight, visceral fat, body mass index, and subcutaneous adipose tissue in overweight and obese people (72–75). The above evidence explains why PWV improvements are better in overweight or obese people who have or carry cardiovascular disease risk factors. Finally, the positive effect of HIIT on PWV was independent of anti-hypertensive medication use, PWV assessment location, and male-to-female ratio. In future studies, it is necessary to expand the study of HIIT for a specific CVD population to find the best personalized program for different CVD populations, and provide a rich theoretical basis and practical examples for the prevention of arterial stiffness in common CVD populations.

We also performed a meta-analysis of AIX, SBP, DBP, and RHR and found that HIIT has no significant effect on AIX but has a significant effect on SBP, DBP, and RHR. Different movement types are purely in dispute with AIX. For example, Ashor et al. (19) concluded that aerobic exercise can effectively reduce AIX in adults. Zhang et al. (20) concluded that long-term aerobic exercise can effectively reduce AIX of patients aged 50–60 years. These results were different from ours. However, a recently published meta-study by Li et al. (22) showed that regular aerobic exercise did not affect the AIX in obese and overweight older adults with or without comorbidities. Participants included in studies by Chrysohoou et al. (39) and Van Craenenbroeck et al. (40) fit the category of obese and overweight elderly persons. Chrysohoou et al. (39) also carried out related resistance exercises in the program, and many studies have shown that resistance exercises have adverse effects on AS (76–79). This may explain the reason why AIX was not significant in our study. Secondly, both articles used disease-related medications during the intervention period, and AIX increased significantly in the HIIT group after the intervention, while no significant change was observed in the control group. Perhaps there was an adverse reaction between some medications and HIIT, which resulted in the statistically insignificant effect of AIX in this study. In the future, more experimental studies are needed to prove whether there are adverse effects of medication use in combination with HIIT treatment. Finally, the small number of studies may also be one of the reasons why AIX is not significant in this article.

Many studies have shown that HIIT can positively affect blood pressure and is superior to MICT (25, 27, 28). We arrived at similar conclusions that HIIT significantly improved blood pressure and RHR in persons with CVD or risk factors for CVD. A decrease of 5 mmHg in SBP or 2 mmHg in DBP was associated with a 14% reduction in stroke mortality and a 9% reduction in coronary heart disease mortality (80, 81). We concluded that HIIT reduced SBP and DBP by 5.43 mmHg and 2.96 mmHg, respectively, with significant implications for reducing CVD mortality. Therefore, HIIT is a viable and effective treatment for lowering blood pressure. RHR is easy to obtain, non-invasive, inexpensive, and can be used as an independent indicator of risk factors and mortality in patients with CVD (82–84), which is more suitable for clinical application than other indicators (84). Reducing RHR may be a therapeutic target in the clinic, and an increase of 10 beats per min in RHR in patients with coronary heart disease is associated with an 8% increase in cardiovascular risk (85). We also conducted a meta-analysis of RHR and found that HIIT significantly reduced RHR by 4.35 beats/min, which reached a significant level. Therefore, HIIT has a positive effect on reducing cardiovascular risk by reducing RHR.

### 4.1 Limitations of the review

This study has some limitations. First, because the literature on HIIT's effect on AS in individuals at high risk for CVD is limited, we cannot consider the effect of HIIT on a particular high-risk population. Second, due to insufficient studies, we cannot conduct a more detailed subgroup analysis of the HIIT protocol, which limits our discussion on the effect of HIIT on arterial hardness in this population. Third, the included studies did not report on adverse events that occurred during the HIIT intervention, which weakened the application of HIIT in persons who were not adapted to high-intensity exercise. Finally, in order to ensure the comprehensiveness of the literature search, we did not strictly limit the population, which also weakened the standardization of the search items in this paper.

## 5 Conclusion

HIIT can improve PWV in persons with CVD or risk factors for CVD. Performing HIIT 2–3 times/week for ≤40 min optimizes PWV improvement. HIIT effectively reduces SBP, DBP, and RHR but does not affect AIX, offering a promising intervention for CVD risk reduction. Sports training is a complex project, and any parameter may have an impact on the exercise effect. Because of the particularity of HIIT exercise intensity, it is necessary to make exercise strategies according to the physical conditions of the participants. Future studies need to focus on designing targeted HIIT interventions for specific cardiovascular high-risk populations to optimize exercise regimens for AS reduction.

## Data Availability

The original contributions presented in the study are included in the article/Supplementary Material, further inquiries can be directed to the corresponding author.

## Supplementary material

### Abbreviations

HIIT, high-intensity interval training; AS, arterial stiffness; CVD, cardiovascular disease; PWV, pulse wave velocity; CF-PWV, carotid-femoral pulse velocity; AIX, augmentation index; SBP, systolic blood pressure; DPB, diastolic blood pressure; RHR, resting heart rate; MICT, moderate-intensity continuous training; RCTs, randomized controlled trials; ACSM, American College of Sports Medicine.
