# Supplementary material for: Effects of high-intensity interval exercise on arterial stiffness in individuals at risk for cardiovascular disease: a meta-analysis
Source: Front Cardiovasc Med. 2024 Apr 17;11:1376861. doi: 10.3389/fcvm.2024.1376861 (PMC11061535; doi:10.3389/fcvm.2024.1376861)
Supplement: Supplementary file 1 [file Datasheet1.docx]

Supplementary table S1

Table 1 Search strategy in PubMed（October 10, 2023）

| Step | Search strategy | Results |
| --- | --- | --- |
| #1 | ("High-Intensity Interval Training"[Mesh]) OR ((((Interval training[Title/Abstract]) OR (Aerobic interval training[Title/Abstract])) OR (Combination training[Title/Abstract])) OR (Intermittent training[Title/Abstract])) | 5293 |
| #2 | ((((Arterial stiffness[Title/Abstract]) OR (Aortic stiffness[Title/Abstract])) OR (Pulse wave velocity[Title/Abstract])) OR (Augmentation index[Title/Abstract])) OR ("Vascular Stiffness"[Mesh]) | 20297 |
| #3 | #1 AND #2 | 110 |
|  |  |  |


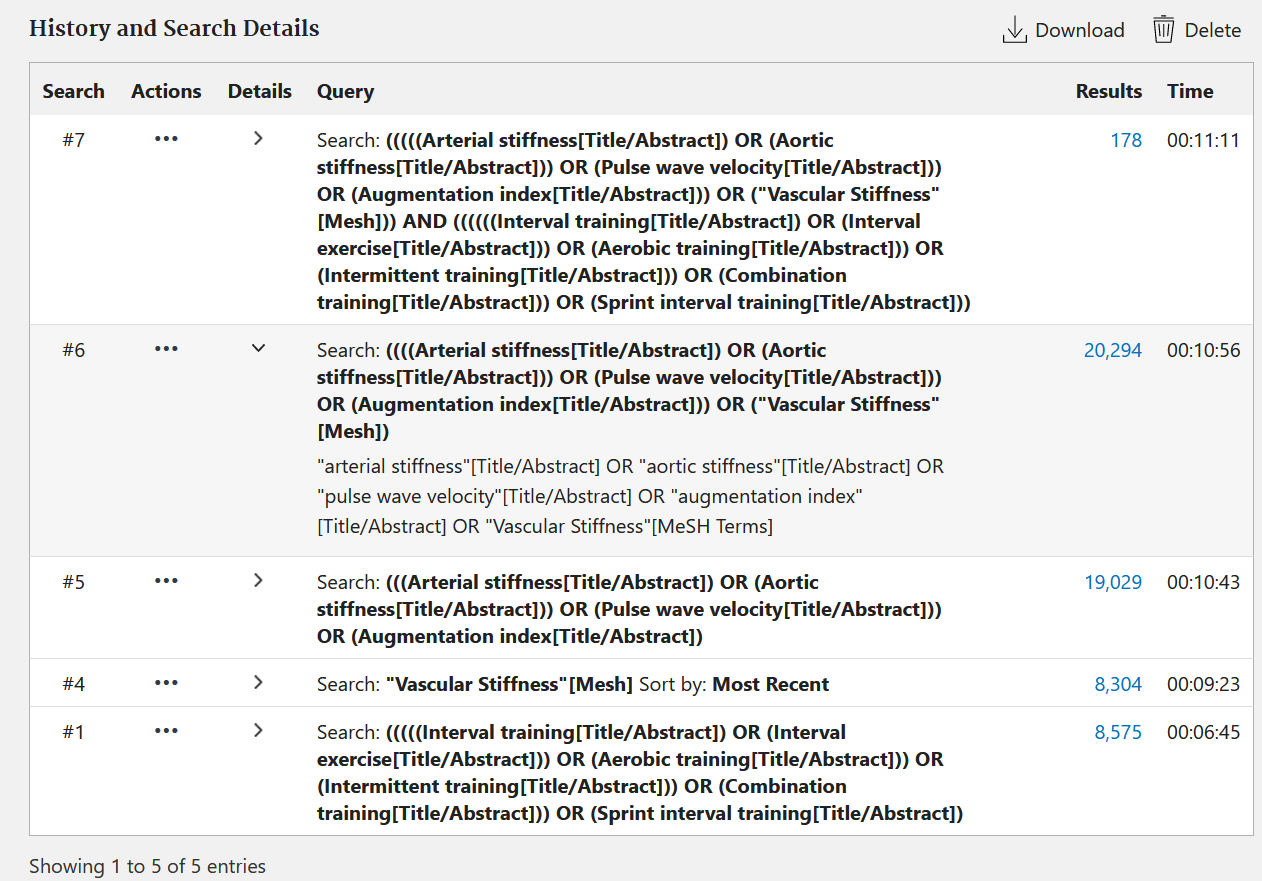


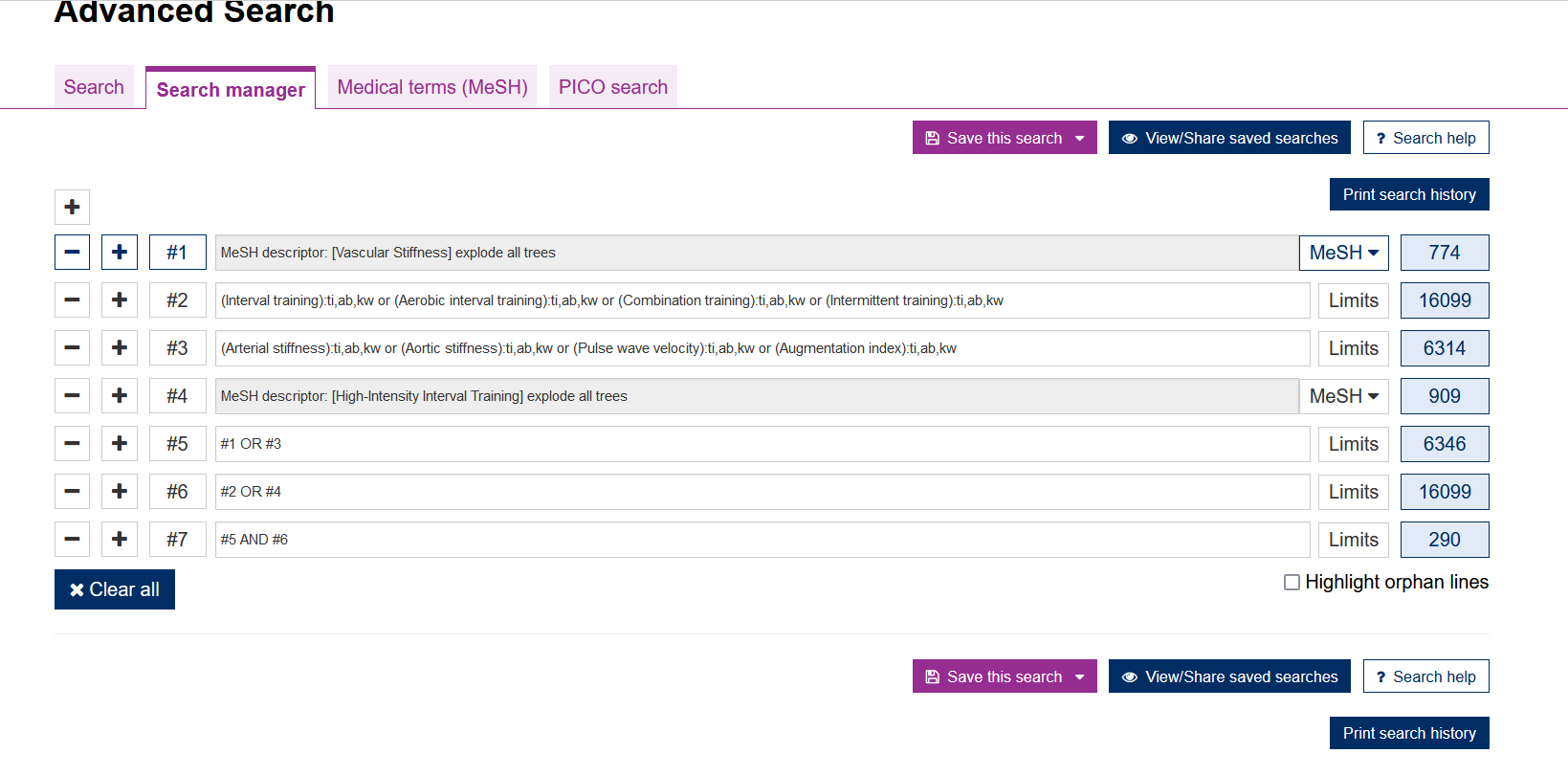


Figure 1 Cochrane search results （*N*=290）（October 10, 2023）


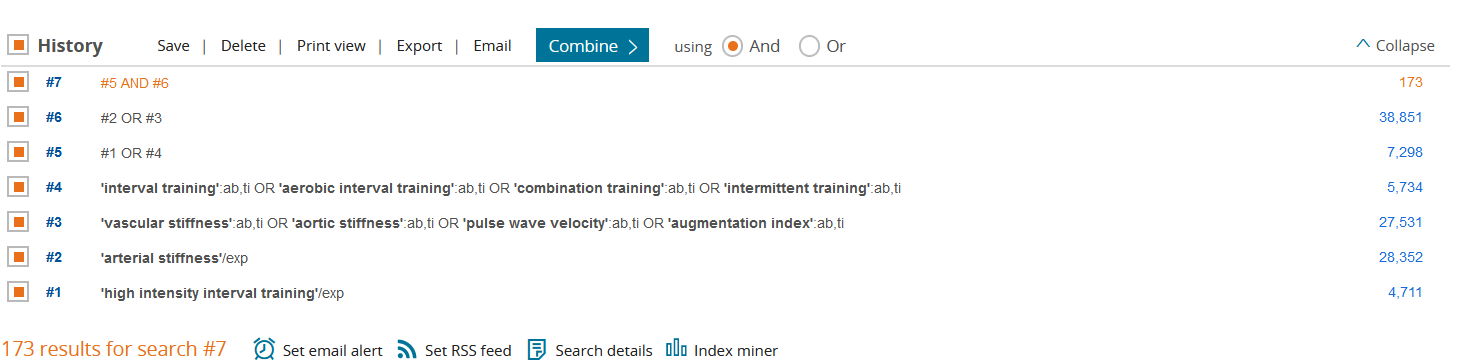


Figure 2 Embase search results （*N*=173）（October 10, 2023）


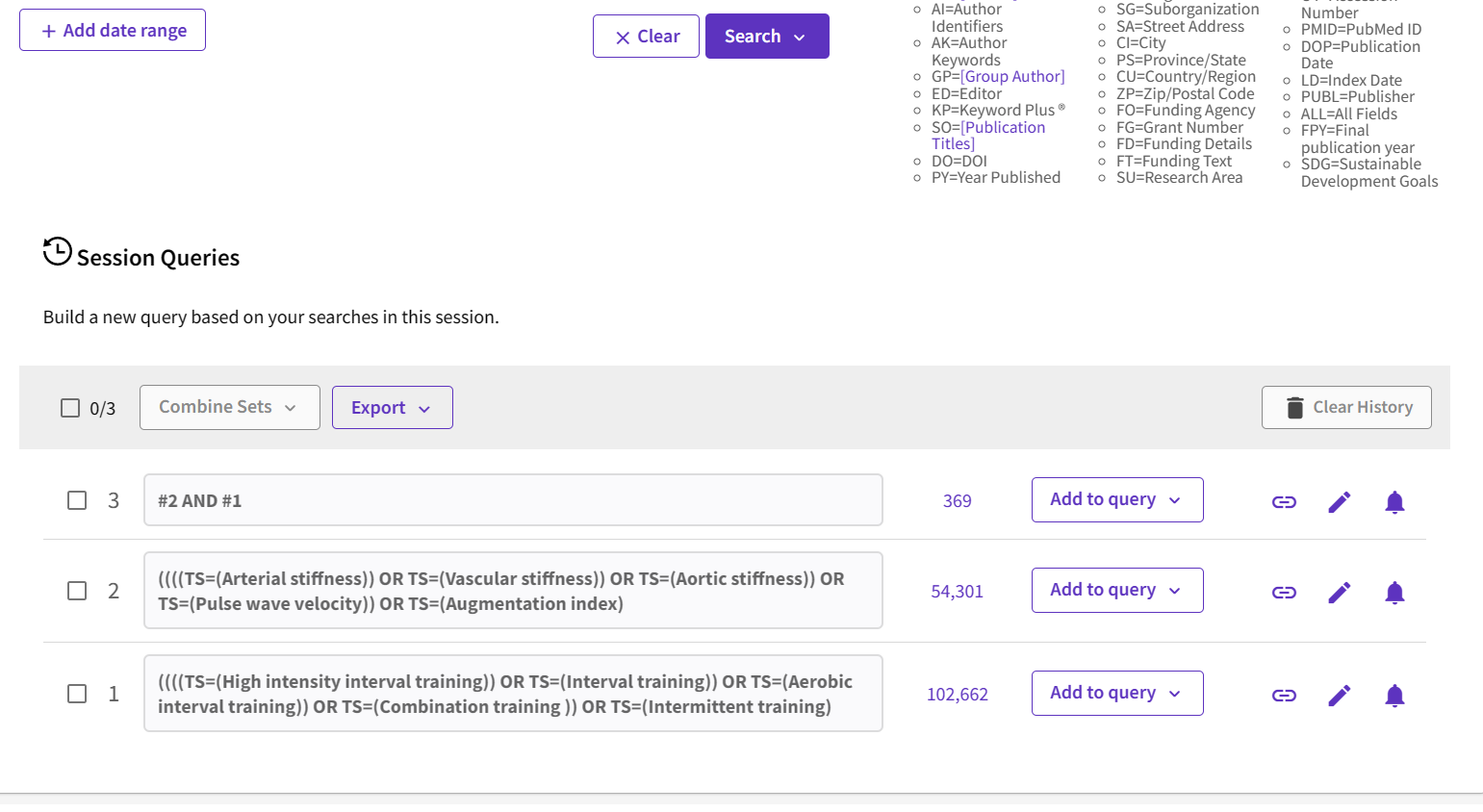


Figure 3 Web of science search results （*N*=369）（October 10, 2023）


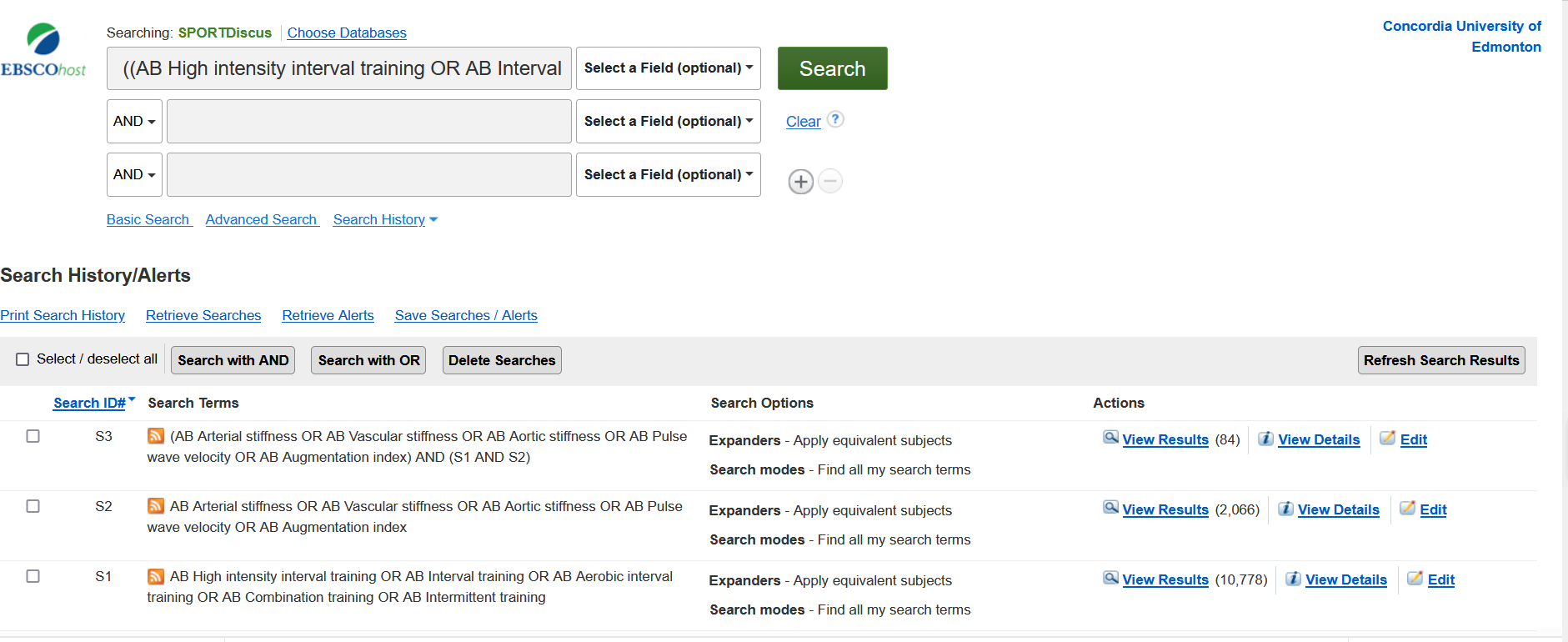


Figure 4 EBSCO search results （*N*=87） （October 10, 2023）

Evaluation of the PEDro scale included in the study

|  | PEDro score | | | | | | | | | | | |
| --- | --- | --- | --- | --- | --- | --- | --- | --- | --- | --- | --- | --- |
|  | 1 | 2 | 3 | 4 | 5 | 6 | 7 | 8 | 9 | 10 | 11 | Total |
| Guimara˜es et al. 2010 | YES | 1 |  | 1 |  |  |  | 1 | 1 | 1 | 1 | 6 |
| Ciolac et al. 2010 | YES | 1 |  | 1 |  |  |  | 1 | 1 | 1 | 1 | 6 |
| Chrysohoou et al. 2014 | YES | 1 |  | 1 |  |  |  | 1 | 1 | 1 | 1 | 6 |
| Van Craenenbroeck et al. 2015 | YES | 1 |  | 1 |  |  |  | 1 | 1 | 1 | 1 | 6 |
| Chuensiri et al. 2017 | YES | 1 |  | 1 |  |  |  | 1 | 1 | 1 | 1 | 6 |
| KIM et al. 2017 | YES | 1 |  | 1 |  |  |  | 1 | 1 | 1 | 1 | 6 |
| Bellia et al. 2017 | YES | 1 |  | 1 |  |  |  | 1 | 1 | 1 | 1 | 6 |
| Mora-Rodriguez et al. 2017 | YES | 1 |  | 1 |  |  |  | 1 | 1 | 1 | 1 | 6 |
| Hanssen et al. 2017 | YES | 1 |  | 1 |  |  |  | 1 | 1 | 1 | 1 | 6 |
| Bahmanbeglou et al. 2019 | YES | 1 |  | 1 |  |  |  | 1 | 1 | 1 | 1 | 6 |
| Bouaziz et al. 2019 | YES | 1 |  | 1 |  |  |  | 1 | 1 | 1 | 1 | 6 |
| Deiseroth et al. 2019 | YES |  |  | 1 |  |  |  | 1 | 1 | 1 | 1 | 5 |
| Ho et al. 2019 | YES |  |  | 1 |  |  |  | 1 | 1 | 1 | 1 | 5 |
| Way et al. 2020 | YES | 1 |  | 1 |  |  |  | 1 | 1 | 1 | 1 | 6 |
| McNarry et al. 2020 | YES | 1 |  | 1 |  |  |  | 1 | 1 | 1 | 1 | 6 |
| TAHA et al. 20203 | YES | 1 |  | 1 |  |  |  | 1 | 1 | 1 | 1 | 6 |

indicate:1:The inclusion conditions of the subjects were clear；2:random allocation；3:Grouping blind；4:The main prognostic indicators were consistent at baseline；5:The subjects were blind；6:Training blind；7:Blind evaluators for at least one major outcome；8:More than 85% of the subjects were measured for at least one major outcome；9:Subjects received treatment or control conditions according to the assignment plan；10:Report intergroup statistical results for at least one major outcome；11:Provide point measurements and variation measurements for at least one major result。
